# Conservation Strategies for Orangutans: Reintroduction versus Habitat Preservation and the Benefits of Sustainably Logged Forest

**DOI:** 10.1371/journal.pone.0102174

**Published:** 2014-07-15

**Authors:** Howard B. Wilson, Erik Meijaard, Oscar Venter, Marc Ancrenaz, Hugh P. Possingham

**Affiliations:** 1 Australian Research Council Centre of Excellence for Environmental Decisions, School of Biological Sciences, The University of Queensland, Brisbane, Australia; 2 People and Nature Consulting International, Jakarta, Indonesia; 3 Centre for Tropical Environmental and Sustainability Science and the School of Marine and Tropical Biology, James Cook University, Cairns, Australia; 4 Kinabatangan Orangutan Conservation Project, Sandakan, Malaysia; SUNY College of Environmental Science and Forestry, United States of America

## Abstract

The Sumatran orangutan is currently listed by the IUCN as critically endangered and the Bornean species as endangered. Unless effective conservation measures are enacted quickly, most orangutan populations without adequate protection face a dire future. Two main strategies are being pursued to conserve orangutans: (i) rehabilitation and reintroduction of ex-captive or displaced individuals; and (ii) protection of their forest habitat to abate threats like deforestation and hunting. These strategies are often mirrored in similar programs to save other valued and endangered mega-fauna. Through GIS analysis, collating data from across the literature, and combining this information within a modelling and decision analysis framework, we analysed which strategy or combination of strategies is the most cost-effective at maintaining wild orangutan populations, and under what conditions. We discovered that neither strategy was optimal under all circumstances but was dependent on the relative cost per orangutan, the timescale of management concern, and the rate of deforestation. Reintroduction, which costs twelve times as much per animal as compared to protection of forest, was only a cost-effective strategy at very short timescales. For time scales longer than 10–20 years, forest protection is the more cost-efficient strategy for maintaining wild orangutan populations. Our analyses showed that a third, rarely utilised strategy is intermediate: introducing sustainable logging practices and protection from hunting in timber production forest. Maximum long-term cost-efficiency is achieved by working in conservation forest. However, habitat protection involves addressing complex conservation issues and conflicting needs at the landscape level. We find a potential resolution in that well-managed production forests could achieve intermediate conservation outcomes. This has broad implications for sustaining biodiversity more generally within an economically productive landscape. Insights from this analysis should provide a better framework to prioritize financial investments, and facilitate improved integration between the organizations that implement these strategies.

## Introduction

Orangutans *Pongo* spp. are severely threatened by habitat loss and hunting [1]–[3] and populations without adequate protection face a dire future [2]. Even for most orangutan populations in areas with legally recognized conservation status, habitat management and law enforcement need to be improved to prevent further population declines [4]–[5].

Two strategies are currently being pursued to conserve wild orangutans: (i) rehabilitating and returning orangutans back into the wild and (ii) preserving current orangutan-populated forest. Rehabilitation centres were initially set up for welfare reasons and as a tool for dealing with confiscated animals held illegally in captivity [6]. In South East Asia, these centres mostly take in animals that have been displaced by deforestation activities [1], [7]. Following rehabilitation, animals are then reintroduced back into their historical range.

As opposed to reintroduction, the management of wild populations is focused on habitat loss and other threats to wild populations, such as hunting. The key to this strategy is to ensure that the quantity and quality of habitat remains sufficient for long-term population viability, without necessarily requiring that an area is legally set aside for conservation. For example, well managed logging concessions provide sufficient resources for orangutans to survive [8], with the revenues from sustainable timber extraction offsetting some of the opportunity costs (i.e. loss of potential revenue) that would occur if the area was fully protected [9].

Reintroduction of orangutans is a widespread strategy that attracts large financial support [10]–[11], but is also being questioned in terms of its contribution to conservation goals [1], [12]. Protecting wild populations also receives substantial investment. Considering that funding for reintroduction or habitat protection is at least partly fungible, planning for optimal conservation outcomes requires that limited funds are allocated wisely. The aim of this study is to investigate how the costs and benefits of a reintroduction strategy compare to those of preserving wild orangutan populations in their natural habitat. We combined GIS analysis and data collated from across the literature within a modelling and decision analysis framework to find the circumstances under which either strategy is optimal.

## Methods

The geographic scope of this study is the two islands of Borneo and Sumatra, South East Asia, currently the only areas with wild orangutan populations. We defined a model in which the forest can be in a number of different states. First, forests are either with or without breeding orangutan populations [3]. Second, forests can be in one of three land uses, i) legal conservation status, ii) production of natural timber (but not industrial tree plantations), or iii) available for conversion to agri- or silviculture (oil palm or industrial tree plantations), but not yet cleared (Table 1). We consider that although industrial tree plantations and oil palm plantations are sometimes used by orangutans [13], these intensive land uses cannot sustain orangutan populations in the long-term and are not considered any further. Third, forests are either with or without “extra protection”, a layer of management specifically to protect the forest for orangutans. These extra protection measures include prevention of illegal logging, fires, and agricultural encroachment, as well as implementing anti-poaching patrols, and human-orangutan conflict management. We considered this protection as an option in all of the forest land uses above including conservation area forest, as the legal land use status of forest is not necessarily related to the quality of forest management and law enforcement regarding orangutans [14], [15].

**Table 1:** Definitions of land management categories considered in this study.

Conservation area	Areas legally gazetted for the conservation of nature and environmental services (National Park, Nature Reserve, Wildlife Reserve, watershed protection, etc.).
Timber Production forest	Any natural forest area legally gazetted for selective timber harvest (no mono-culture timber species, clear cutting or conversion to agriculture).
Conversion forest	Forest areas not yet cleared but ultimately slated for conversion for agricultural uses, such as oil palm, or silvicultural use such as softwood plantations.

Our study looked at two different ways of maintaining the number of wild orangutans. The first was to provide extra protection for wild orangutans in their forest habitat (strategy P). This can be done in: (i) conservation area forests (provide extra protection only); (ii) timber production forest (introduce reduced impact logging practices, for example as prescribed by the Tropical Forest Foundation and under the Forest Stewardship Council certification schemes [16], and also provide extra protection); or (iii) forest available for conversion to agriculture (purchase forest and provide extra protection). We show in the results section that due to the high opportunity costs, working in conversion forest was never the most efficient strategy, so we don't present further details here. The second strategy was to rehabilitate orangutans that have been rescued from captivity or forest under conversion, and reintroduce them into orangutan-free forest and then provide extra protection (strategy R). Extra protection is needed for the release site otherwise the same processes of hunting and illegal logging will wipe out reintroduced orangutans [17] just as it does for current wild populations [18]. A third strategy, keeping orangutans in permanent captive conditions, makes no direct contribution to the survival of wild orangutans so is not discussed further.

For both strategies, the conservation objective was to maximise the number of wild orangutans alive at a specific management time horizon, *t_H_*, for a given budget. All effects later than the time horizon were not considered. A particular time horizon may be chosen as conditions may significantly change after this point in time (for example, Indonesia has made a legally binding decree to empty, through reintroductions, all rehabilitation centers by 2015, and to protect all wild populations by 2017 [19], although it is difficult to see how these goals will be achieved given current conditions). We assess the effectiveness of previous conservation policies at this time horizon.

The spatial variation of orangutan abundance depends on forest types and stages [20], and the degree of hunting. In our models, we used an average orangutan density calculated across this variation in density (see *Parameter estimates* below). The number of orangutans then equalled the number of hectares of orangutan populated forest multiplied by the average density of orangutans per hectare. Our goal of maximizing the number of wild orangutans is equivalent here to maximizing the total number of hectares of any forest type with orangutans present (without regard to whether they have extra protection or not). Later we include a higher orangutan population growth in forests with the extra layer of management, which resulted in a different density of orangutans in different forest types. We also return to this issue of spatial variation in density in the discussion.

### Conservation forest only

Our simplest model includes only forest that is already legally, but not effectively, conserved. We assumed that: 1) there was always enough conservation forest free of orangutans for reintroduction (see File S1 for justification, although relaxing this assumption does not qualitatively change our results); 2) there were always enough orangutans in rehabilitation centres for reintroduction (see File S1); and 3) there was enough orangutan-populated forest for protection (we relax this in the next section). We modelled the amount of conservation forest with orangutans but without extra protection, *CF*, and the amount of conservation forest with both orangutans and extra protection, *CF_p_*:
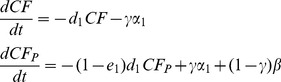
(1)
*CF* and *CF_p_* are functions of time and *CF_p_* at time 0 equals zero, *d_1_* is the rate of conservation forest loss, *e_1_* (0<*e_1_*<1) is the efficiency of extra protection in reducing the rate of forest loss (*e_1_* = 0 is when extra protection provides no benefit in reducing the rate of forest loss, and *e_1_* = 1 is when there is no loss of forest with extra protection), *α_1_* is the total potential amount of conservation area that can be protected per year if all the budget was used for this purpose, *β* is the total potential amount of orangutan-free conservation forest that can be converted to protected forest per year by reintroducing orangutans (both *α_1_* and *β* have units of hectares per year), and *γ* is a control parameter that changes the proportion of the total budget spent on the two strategies (a proportion *γ* is spent on protection, P, and [1- *γ*] is spent on rehabilitation and reintroduction, R). The parameter *α_1_* equals *B/C_P_*, where *B* is the total budget per year and *C_P_* is the cost of extra protection per hectare of forest, and *β = B/C_R_*, where *C_R_* is the reintroduction cost per hectare (i.e. rehabilitation cost per orangutan plus the cost of forest protection after release per orangutan all multiplied by the average orangutan density per hectare).

### Parameter estimates

We determined the area of Indonesian and Malaysian forest housing orangutans in 2010 by overlaying, through geographic information system analyses, a map of 2010 forest cover [21] with a map of the distribution of the Sumatran and Bornean orangutans [22]. We determined the area of this forest that was currently under some form of conservation management by overlaying a map of IUCN category I–VI protected areas [23]. We find that, in 2010, there was a total 12,177,153 ha of forest within the mapped range of orangutans, of this, 3,398,392 ha are in some form of conservation management.

Regardless of the legal status of land in Indonesia and Malaysia, forests are being lost due to unsustainable logging, anthropogenic fires and agricultural conversion. We calculated the rate at which forests are being lost by determining the proportion of forest in these countries that was cleared, or otherwise transitioned to non-forest, between 2000 and 2010 [21] using high resolution land cover maps for this period [21]. These maps are not able to distinguish between regenerating natural forests and industrial timber plantations. Our estimate of forest loss therefore represents the annual percent conversion of all forest types (primary, regenerating and plantation) to non-forest land covers. As we had data from two time periods only, we were unable to establish whether habitat loss is a linear process, or follows an alternate trajectory. This data source is currently the highest quality land cover data available for Indonesian Borneo and Sumatra, with validated accuracy for 2000 for forest/non-forest of 91.7%, and for 2010 forest/non-forest 93.6%. We found that the rate of forest loss was lower in conservation forest (16,487 ha yr^−1^, or 0.485% yr^−1^), than in non-conservation forest (164,949 ha yr^−1^, or 1.879% yr^−1^).

To estimate the ongoing management costs of effectively protecting forest, we used data from McQuistan *et al.* (2006) [24]. We extracted the optimal budget of effectively managing a strict no-take protected forest area. The cost of managing forest that is currently under legal conservation status was used to be the optimal per hectare budget for a national park [24] in Indonesia, and the cost of managing forest that is available for conversion was taken as the optimal budget for a forest park [24]. We stress that the cost figure is not what is currently being spent, rather it is an estimate of the optimal budget required to make sure these parks are effectively managed to fulfill the park's objectives. It's the best case scenario cost for effective forest protection, i.e. for *e_i_ = 1*. Total forest protection can, and has been achieved in practice: in Kalimantan the Wehea protection forest has had zero forest loss between 2004 and 2014; as has the Sungai Wain protection forest between 2000 and 2014; in Malaysian Borneo the Danum Valley, Tabin Wildlife Reserve, and Sepilok forest have had no forest loss. Hence achieving *e_i_ = 1* is possible, although it is probable that protection would be less than 100% effective in many, or most, cases. We have estimated the loss rate of legally protected forests to be one quarter the loss rate of legally unprotected forests (0.486% vs. 1.88%, above). We have used this as a guideline for the efficiency of our extra layer of forest management, and have used *e_i_* = 0.75 (i.e. a reduction in forest loss of 75%).

Management costs of effectively protecting forest were calculated as the amount of money needed today to fund all the future costs of management up to the time horizon. This uses a discount rate for future costs. Costs were estimated by an initial setup cost ($52.4/ha) and an ongoing management cost ($3.87/ha) [24], and a discount rate of 10% (a value typically used by Indonesian companies, [25]). All costs have been converted to 2010 US$. One should note that *C_P_* and *C_R_* are dependent on *t_H_* (the time horizon), although as *t_H_* increases, then this dependence is very small (*t_H_*>20).

The costs of protecting forests available for conversion to agriculture were taken from [26]. These figures were based on a literature review of the revenues derived from intensive logging, and from the financial reports of oil palm companies. We assumed that logging and clearing of the land would take place over five years, and that oil palms take five years to reach maturity (as in [26]). The opportunity costs of purchasing these forests were estimated as the net present value of the annual profits discounted at a rate of 10% per annum.

Rehabilitation and reintroduction costs (*β*) have been estimated from the operating costs of the Borneo Orangutan Survival Foundation (BOS), the largest primate rescue and rehabilitation organization in the world. The operating budget for BOS in 2007 was $4,322,026 [27], or $5,403 per orangutan. When calculating the total cost of rehabilitating an orangutan, we estimated that the cost of releasing an orangutan into the wild is $5,000, and 10% of captive orangutans would be released after one year, a further 20% after two years, 30% after three, 20% after four and 10% after five. We assumed that the remaining 10% of orangutans would never be fully rehabilitated and would remain in captivity for the duration of their life. After release, we assumed that rehabilitated orangutans would suffer a 50% mortality rate [7]. From these parameters, the average cost per successfully released orangutan is $44,121. In addition, there was the cost of extra forest protection after release per orangutan (the same as for the P strategy).

The average orangutan density across the two islands was estimated to be 42 ha/animal, which is a population size weighted average for the densities of Sumatra (25 ha/animal) and Borneo (44 ha/animal). The average density from each island was based on the mid-point of the densities found on those islands: 1–7 animals/km^2^ for Sumatra; and 0.5–4 animals/km^2^ for Borneo [20].

### Conservation and timber production forest

It is always more cost efficient to reintroduce orangutans into conservation forest rather than timber production forest (see costs in Table 2). For P, the cost of providing extra protection for timber production forest was higher than for conservation areas, and there was an extra cost for introducing reduced impact logging practices that maintain forest structure. However, there was also an advantage of protecting timber production forest, as a higher rate of forest destruction could be prevented. The dynamics of the four kinds of forest (conservation forest, no extra protection; production forest, no extra protection; conservation forest, extra protection; and production forest, extra protection) are described by the equations:
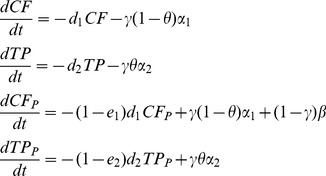
(2)where *TP* is timber production forest populated with orangutans, *TP_P_* is timber production forest populated with orangutans with extra protection, *d_2_* is the rate of timber production forest loss, *α_2_* is the total potential amount of timber production forest that can be protected in any one year if all the budget was used for this purpose, *e_2_* is the efficiency of extra protection in reducing the rate of timber production forest loss, and *θ* is a control parameter that changes the proportion of the P budget spent on conservation or timber production forest. There is a cost in timber production forest for introducing sustainable logging practices ($20.9 ha^−1^, [28]), and the extra protection costs per hectare are also higher ($16.86 ha^−1^ yr^−1^ versus $3.87 ha^−1^ yr^−1^ for conservation forest). All other parameters and states are as in model 1. The costs of implementing reduced impact logging practices are not the full opportunity costs of conventional logging, as these practices usually produce a similar timber yield to conventional logging practices [29]. Instead, the cost presented in Table 2 is the additional cost of pre-harvest planning, vine cutting, felling, skidding, supervising and training associated with reduced impact logging. A key point is that these logging practices are not introduced into primary forest areas, rather they are introduced into areas that are already being logged and will continue to be logged.

**Table 2:** The parameter estimates, probable range of values, and the critical value at which the optimal strategy changes.

*Parameter*	*Estimated value – conservation forest (range)*	*Critical value -with hunting and orangutan popn growth (no hunting or popn growth)*	*Estimated value – timber production forest (range)*	*Critical value- with hunting and orangutan popn growth (no hunting or popn growth)*
Time horizon, (yrs)	50	12	50	25 (52)
	(5–100)	(49)	(5–100)	(52)
Rate of forest loss (yr^−1^), *d_i_*	0.00485	never (0.0044)	0.0188	never (0.0196)
	(0.0046–0.0052)		(0.0173–0.0203)	
Protection management cost	94.6	459.4 (103.4)	257.2	420.0 (250.0)
(US$ ha^−1^)	(81.5–126.6)		(202.4–396.5)	
Opportunity cost (US$ ha^−1^)	0	N/A	20.9	176 (13.1)
			(9.6–32.3)	
Efficiency of protection, *e_i_*	0.75	0.22^a^ (0.69)	0.75	0.52 (0.76)
	(0.5–1.0)		(0.5–1.0)	
Rehab cost, ($ orangutan^−1^)	44,121	9,124 (38,705)	44,121	26,900 (45,500)
	(33,091–55,151)		(33,091–55,151)	

The optimal strategy using the estimated values was protection. The critical value at which reintroduction resulted in more wild orangutans than protection was calculated by keeping all the other parameters constant at the estimated values, and then varying one parameter to find when the optimal strategy changed. Values were calculated by simulation (although the formula *t_H_≈2C_P_/d e C_R_* can also be used as an approximation to the critical point). The protection cost has three underlying parameters that were varied; the initial setup cost, the cost per hectare, and the discount rate. For clarity, we have summarized these into variation in the overall protection cost. Hunting was assumed to result in a loss of 0.485% p.a., population growth was 0.75% p.a., and a budget of $5M p.a. was used.

a when the efficiency is <1, we assumed that the orangutan growth rate was *e_1_* * 0.75%.

### Hunting

Hunting of orangutans, especially in Kalimantan, is a major threat [1], [18] and we assume that nearly all orangutan populations are below carrying capacity because of past hunting [30]. We modelled hunting by including extra loss terms for the dynamics of non-protected, orangutan-populated forest (*-h_1_CF* and *–h_2_TP* in the equations for *dCF/dt* and *dTP/dt*). Recent studies have indicated that the rate of loss of orangutans due to hunting is of a similar order of magnitude as to forest destruction [18]. Here we have used a conservative estimate of hunting as being of a similar order to the loss of conservation forest (0.485% p.a).

### Orangutan population growth

Some natural repopulation of the forest by orangutan population growth in well managed (i.e. with extra protection) forest would be expected as current population levels are probably lower than carrying capacity [30]. We modeled this by including an orangutan growth rate, *r*, in forest with extra protection only. This growth rate thus represented an increase in the density of orangutans within these areas, as opposed to a colonization rate of new areas. We kept track of when each area of forest received extra protection, and the density of orangutans in these areas was multiplied by a factor *r^t^*, where *t* was how long it has been protected. Orangutan populations under no external threats can grow at a maximum of 2% annually, although very few wild populations probably achieve this maximum theoretical rate [31]. We used a more biologically reasonable growth rate of 0.75% p.a.

### Leverage

A possible extra benefit of the rehabilitation and reintroduction strategy is that it attracts media attention, thereby raising awareness about the orangutan's plight, putting pressure on conservation authorities, and providing fund raising opportunities. In this case, instead of a set budget amount being split between the two strategies, the more reintroduction is used as a strategy then the bigger the budget. We modelled this by assuming the total budget increases by a factor *l*, in proportion to the fraction of the reintroduction strategy being adopted; *B_T_ = γ B+(1−γ) l B*, where *γ* is the proportion of the budget spent on P.

### Parameter error estimates

It's possible that some institutions might have short-time horizons dictated by funding or voting cycles, whilst others might consider perpetuity as the only appropriate horizon for biodiversity conservation. To reflect this uncertainty we chose a range for the time horizon of 5–100 years.

For the range of the rate of forest loss, we used the maximum errors in the land cover maps (8%, [21]). We don't have any a priori reason to think that errors will be biased on one direction or another, so a pixel that is thought to be forest is as likely to be non-forest as a pixel that is thought to be non-forest is to be forest. In general a land cover classification algorithm will be established such that it equalizes the rates of false negatives and positives, and thereby minimizes the overall error rate. If there is no bias in the error, then the land cover errors will tend to cancel out.

The range for the protection cost was estimated by varying the initial setup cost, the cost per hectare, and the discount rate separately by ±25% each to find the maximum possible range. This fits our practical experience of the costs of protective management in different parts of Indonesian and Malaysian Borneo. Also, upfront costs tend to be higher than long-term maintenance costs, and the 25% variation captures this adequately. The budget for extra protection is the estimate for what is needed for full and effective protection, so the efficiency should be close to 1. However, we took a precautionary approach and analyzed a range between 0.5–1.0. The cost for implementing reduced impact logging was varied from $9.6–$32.3 (±54%, [28]). Finally, for the costs of rehabilitation and reintroduction we did not have any data that would allow us to estimate variability. Consequently, we varied the reintroduction cost by ±25%, the same as for the protection costs.

### Sensitivity analysis

All the parameters used in the above models have been systematically varied. We primarily looked at how variation in the parameters affected our analytical results, but we also checked our analytical results against simulations. We performed five different sensitivity analyses on the parameters. First, we varied a single parameter at a time. Second, we randomly selected a value for each parameter from their range and then calculated whether protection or reintroduction was the best strategy. This was done 50,000 times to find the probability of each strategy being optimal, and to sample the whole parameter space. Hence there was no need for a complex sampling technique. Third, one particular parameter was selected and its value fixed, whilst selecting random values for the other parameters. This was done 50,000 times to find the mean strategy probabilities for a fixed value of the chosen parameter. We then systematically selected different values for the chosen parameter across its range to find how the optimal strategy changes. This approach demonstrated how sensitive the optimal strategy was to variation in each parameter, averaged across the sensitivity in the response to all the other parameters. Fourth, during each simulation, we randomly selected a new value for each parameter from their range every year. This was done 50,000 times to find the probability of each strategy being optimal when the parameters are randomly changing with time. Fifth, an alternative to a fixed time horizon is to allow uncertainty in the end point, as certain knowledge of when conditions might change is rare. We investigated this by using a fixed probability that in any one year the model ends. If this probability was 0.05, this would equate to an average time horizon of 20 years. We then ran 1,000 simulations of the model (each will have a different end time) and determined how well each strategy performed in terms of the number of wild orangutans alive at the end averaged over all the simulations.

We looked at pursuing either a single strategy (P or R), a mixed strategy (a fixed proportion of each) for the whole time, or switching between the two strategies over the time horizon (in which case the control parameter, *γ*, above is a function of time).

## Results

### Conservation forest only

**Figure 1:**
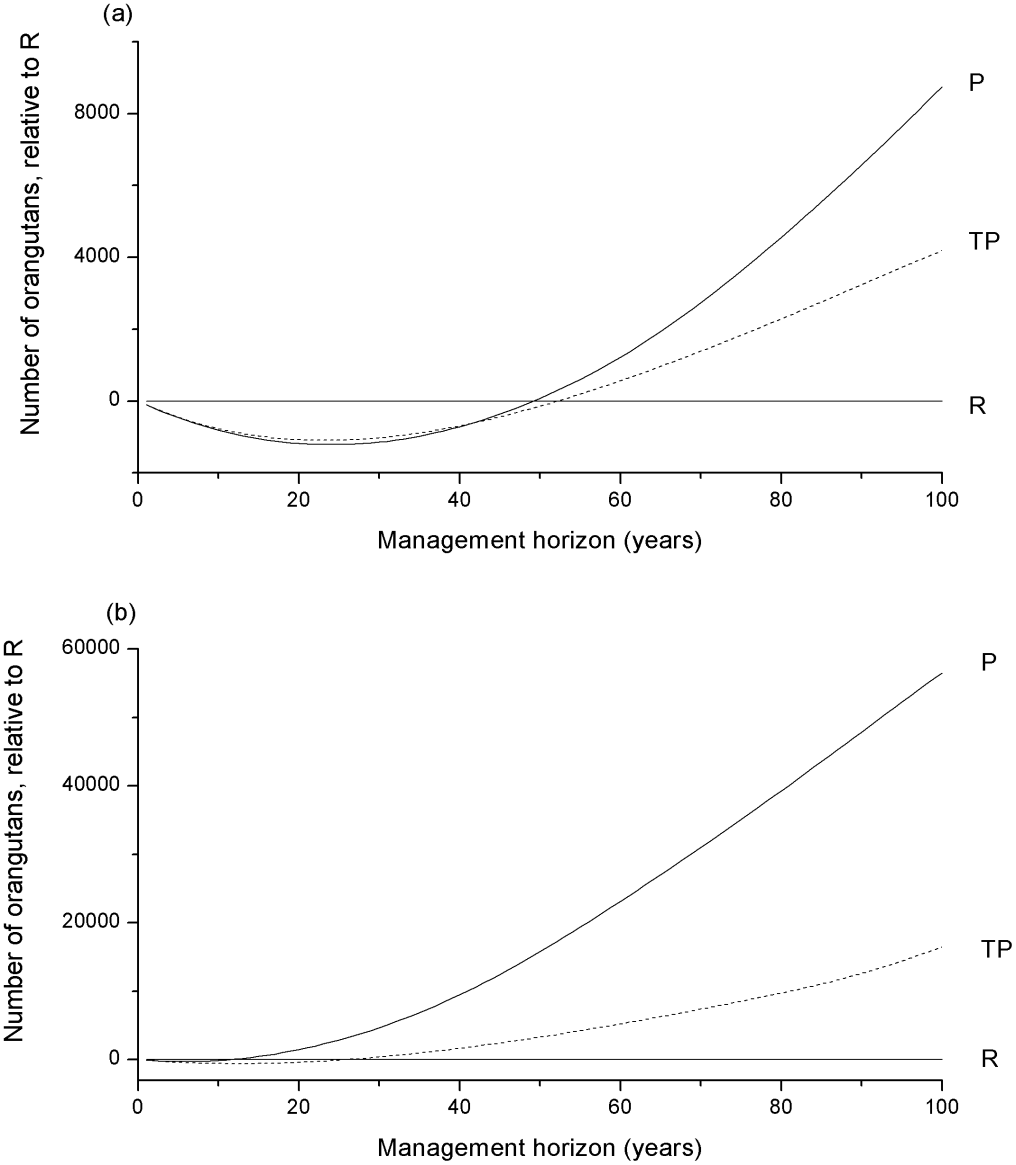
The relative performance of different strategies. The y-axis shows the difference between the number of orangutans for each strategy relative to the R strategy (with reintroduction into conservation areas). R compared to itself is a straight line at zero, above zero a strategy performed better than R, below zero a strategy performed worse than R. The strategy P protects conservation areas first. The strategy TP introduces sustainable logging and protection into timber production forest first. The budget was $5M per year, other parameters are in Table 2. (a) Without hunting or orangutan population growth. The critical time horizon is 49 years for P, and 52 years for TP. (b) Hunting and orangutan population growth included. The critical time horizon is 12 years for P, and 25 years for TP.

The total number of hectares of forest populated by orangutans at the time horizon, *CF_T_(t_H_)*, was found by integrating model (1) with respect to time and summing *CF* and *CF_p_*. The strategy that maximised *CF_T_ (t_H_)* is also the one that maximised the number of wild orangutans, as the two are proportional. This maximum can be determined by differentiating *CF_T_ (t_H_)* with respect to *γ* (see File S1). As this is linear with respect to *γ*, then the optimal solutions occurred at the limiting values for *γ* and there was no optimal mixed strategy which would have allocated a proportion of the budget to both strategies. More complex solutions might occur if there were greater complexities in the cost functions, *C_P_* and *C_R_* (e.g. the cost of extra protection per hectare decreased as the amount of forest protected increased). Using our estimates of the parameters (Table 2), the optimum was *γ* = *1* (P). However, different parameter values resulted in a different optimal strategy. The critical point at which the optimal strategy changed was when *t_H_≈2C_P_/d e C_R_* (found by solving *d CF(t_H_)/dγ = 0*, see File S1). Rearranging this equation for any particular parameter will give the critical value for that parameter (Table 2). The critical time horizon was approximately 49 years. We have verified this analytical result using numerical simulations (see Fig. 1a). For time horizons shorter than this, the optimal strategy was to allocate the entire budget to R. For longer time horizons, the optimal strategy was to spend the entire budget on P. We emphasise that the critical time at which the optimal strategy changes does not represent a time at which to switch strategies, rather if a timescale longer that 49 years is considered, P represents the best strategy adopted for the whole time; for a shorter timescale, R is the best strategy.

### Protecting conservation or timber production forest

Analytical work (see File S1) shows that the optimal protection strategy is to concentrate 100% of the resource allocation into protecting the one forest type that delivers the most benefit (in terms of the number of wild orangutans) for a fixed cost; i.e. there is no optimal mixed protection strategy. The optimal strategy is the one which has the highest value of the quantity *φ_i_ d_i_ e_i_/C_i_*, where *φ_i_* is the density of orangutans in forest type *i* relative to pristine forest. The best forests to protect are ones where there is a high density of orangutans, where there is a high rate of destruction in the absence of protection, high efficiency of protection, and low cost. Frequently flooded lowland swamp forest with low agricultural potential would be an example.

Protecting timber production forest was more cost-effective than providing protection for conservation forest (*φ_i_d_i_ e_i_/C_i_* = 4.8e-5 and 3.8e-5 respectively). However, our analytical results used an approximation, *O(d_i_^2^t^3^)≈0* (see File S1), which was less accurate at long time horizons or when there were high rates of forest destruction. Simulations confirmed that timber production forest was more cost-effective only for shorter timescales (less than 40 years, see Fig. 1a, and less than 55 years if *e_i_ = 1*), whereas for longer timescales extra protection for conservation forest is more cost-effective.

Purchasing conversion forest that would otherwise be converted to silviculture and agriculture results in a high opportunity cost of removing potential timber revenues (estimated to be $2,268 ha^−1^, [9]) and agricultural rents (estimated to be $6,766 ha^−1^ for oil palm, [9]). This means the product *φ_i_ d_i_ e_i_/C_i_* ( = 5.3e-6 using the opportunity cost for timber revenues only) was an order of magnitude lower as compared to protecting other forest types and the purchase and protection of conversion forest was never a cost-effective solution as compared to protection of the other two forest types.

### Hunting and wild orangutan growth rate

Hunting has structurally the same effect in the models as an increase in the value of *d_i_*, the rate of forest loss. Hence, including hunting made the protection strategy more likely to be optimal. If the loss of orangutans due to hunting is of a similar order of magnitude as for forest destruction, as recent studies have indicated [18], then the critical values of other parameters were significantly changed (Table 2). Inclusion of the orangutan growth rate had a similar effect; the protection strategy was more likely to be optimal and the impact on critical values was significant (Table 2). Inclusion of both effects resulted in the critical time horizon dropping to only 10 years in conservation forest only, and to 18 years in timber production forest. Simulations showed that protection of conservation forest is always more cost-effective than protection of timber production forest when an orangutan population growth rate was included, with or without hunting (Fig. 1b).

### Leverage

Leverage favoured the reintroduction strategy, approximately in proportion to the increase in the budget for reintroduction. A 50% increase in the budget (*l = 1.5*) resulted in a 50% increase in the critical time horizon. For long time horizons approximately greater than 50 years (which favoured protection), and with a budget approximately twice as large for reintroduction (*l>2*), a mixed strategy of both protection and reintroduction was optimal. The proportion of the budget spent on each depended on the time horizon (as it increased then the proportion of the budget spent on protection increased), and the degree of leverage (as leverage increased then the proportion of the budget spent on reintroduction was higher).

### Sensitivity analysis

**Figure 2:**
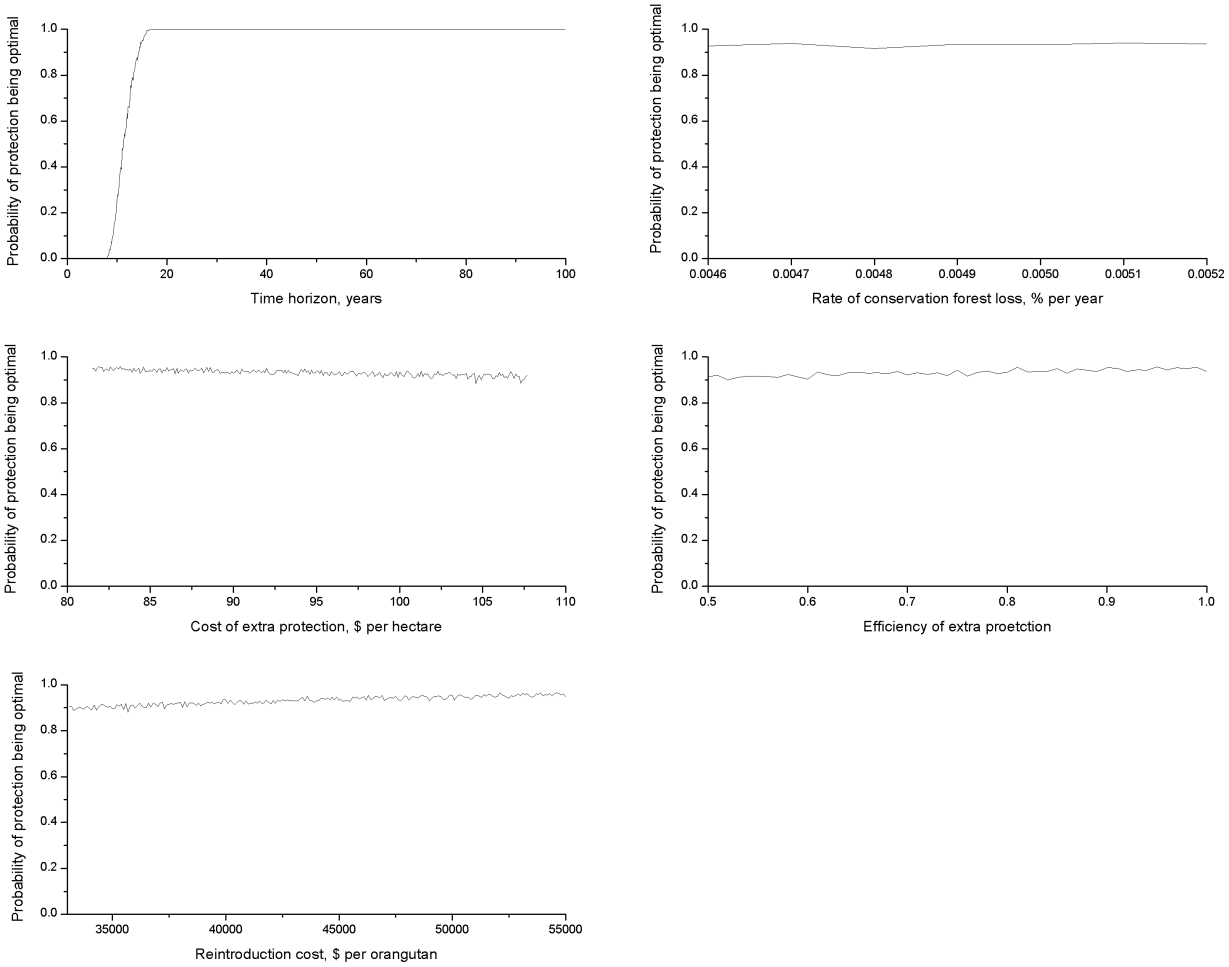
Sensitivity analysis. Each figure shows the probability of protection being the best strategy, when holding one parameter fixed whilst varying all the others. The x-axis gives the fixed value of the parameter in question, all other parameters were randomly chosen from their range. The y-axis is the probability of protection being the optimal strategy, averaged over 50,000 random selections. When every parameter was allowed to vary randomly, the probability of protection was 0.93. The parameters are for conservation forest (see Table 2), with hunting and population growth included.

We performed five different sensitivity analyses. (i) Varying one parameter at a time. Analysis of the parameters showed reintroduction was more likely to be the optimal strategy if: (a) the time horizon was small; (b) the rate of forest destruction *d* was small; (c) the cost of protection was high; (d) efficiency of protection was low; or (e) the cost of reintroduction was low (see Table 2 for the critical values). However, inspection of the range of possible parameter values showed that only a short time horizon can result in reintroduction being the optimal strategy when hunting and population growth are included. All other critical values were outside the estimated range, typically by a considerable amount. (ii) When all the parameters were allowed to vary randomly, the probability of protection being the optimal strategy was 0.93 (with hunting or population growth) and 0.53 (no hunting and population growth). (iii) When one parameter was fixed and the others chosen randomly, only the time horizon significantly influenced whether protection or reintroduction was the optimal strategy (Figure 2). (iv) Randomly changing the parameters in every year produced very similar results to fixing the parameter at the estimated value. (v) When there was variability in the end point, the qualitative results were the same as before; although protection being the optimal strategy occurred at shorter timeframes than previously. As for fixed time horizons, there was no optimal mixed strategy.

## Discussion

Protection of forest is a long term strategy for conserving orangutans. Reintroduction seeks to increase the wild population of orangutans via re-establishing viable populations in areas where they have vanished. However, it is approximately twelve times more expensive than protection (per orangutan), which means less forest can be protected for the same budget, and so a short-term gain occurs at the expense of more forest and orangutan loss in the future. In effect, for long time horizons prevention is better than cure. These results show that the timescale over which conservation goals are being assessed is critical to understanding what type of management approach is cost-effective [32]–[33]. When all the effects we studied were included (hunting, orangutan population growth, leverage), protection is a more cost-effective strategy when the timescale is greater than 10 (no leverage for reintroduction) to 20 years (the budget for reintroduction is twice that of protection). This is a timescale short enough to be realistic and relevant for organisations working in the field.

For both strategies, (reintroduction and habitat protection), maximum long-term cost-efficiency is achieved by working in conservation forest. This means that a proper network of protected areas remains the ultimate goal for long-term orangutan protection. However, introducing reduced impact logging practices coupled with additional protection for orangutans in timber production forest is a strategy intermediate in performance between reintroduction and protecting conservation forest, and in some cases can outperform protecting conservation forest (at intermediate time scales when there is a high efficiency of protection). Timber production forest is more expensive to effectively protect per hectare, but there is a benefit as a relatively higher rate of destruction of orangutan habitat can be prevented. This is similar to work in conservation planning where prioritising areas is a combination of conservation value (how much we stand to lose) and threat (how likely we are to lose it without intervention) [34]–[35].

Although conservationists and the public are generally keener to protect what is perceived as vast and genuine patches of wilderness, our results reveal that there may well be a role for well managed production forests for orangutan conservation in some instances. We recognize that opening up forests for timber exploitation or other types of industry brings people, roads and infrastructure into orangutan habitat and results in increased poaching [36]. However, we are not suggesting that the reduced impact logging practices be introduced into primary forest areas, rather we've considered their introduction into areas that are already being logged. Orangutans are hunted for food throughout their range, especially in the areas where the commercial timber industry operates [18], [37]. Orangutans would be hunted in these areas irrespective of whether conventional logging or reduced impact logging would be implemented. Reduced impact logging would in fact have a better chance of reducing hunting as compared to conventional logging because of the often related requirements to close up skid trails and logging roads. There would also be additional advantages if sustainable forest management certification was sought, through organizations such as the Tropical Forest Foundation (TFF) or the Forest Stewardship Council (FSC). For the FSC certification in particular, timber extraction needs to comply with a series of non-harvesting related practices that are likely to reduce hunting pressure on orangutans, such as control of illegal hunting in the concession area, wildlife monitoring and community development programs [16]. Furthermore, certification would allow a premium to be fetched for the timber produced. Overall, sustainable logging practices in concessions that enforce a zero-killing policy are compatible with the maintenance of viable orangutan populations [8].

We have addressed uncertainty in our parameter estimates, parameters changing through time, interactions between parameters, and stochastic endpoints. This systematic look at parameter variation showed that the time horizon was the most significant parameter influencing which strategy was optimal. An explanation for this lies in the analytical result for the critical values. How the optimal strategy changes is linear with respect to all the parameters, so that the parameter elasticities (the ratio of the percentage change of the result with respect to the percentage change in the parameter) are all equal to one (so that a 10% change in a parameter will change the result by 10%). Hence the parameter which varied the most (the time horizon), was the most important in influencing the result. Interaction effects between the other parameters did not significantly influence the optimal strategy, as when all the parameters were randomly varied, the probability of protection being the optimal strategy was 0.93 (with hunting and population growth) or 0.53 (without hunting and population growth). This value is dominated almost exclusively by whether the chosen time horizon was above or below the critical value of 12 and 49 years (randomly choosing from the range 5–100 years resulted in *t_H_*>12 years 93% of the time and *t_H_*>49 years 53%), despite potential interactions between other parameters. Finally, if hunting and the orangutan population growth were included, changes to our estimates of the parameters (apart from the time horizon) would have had to be very large (greater than 75%) to influence the optimal strategy (Table 2).

One uncertainty, environmental variability or catastrophes, was not analysed as population viability modeling done for orangutans have assumed that severe climatic events, such as very dry El Niño years, could kill up to 3.5% of all orangutans [31]. This is similar to the annual combined losses due to hunting and forest destruction already included within our model (Table 2), yet the environmental catastrophes are rare. Hence they are unlikely to be significant relative to other threats.

Two issues we have not addressed in this paper are leakage and spatial variation of orangutan abundance. In our model, leakage (where protection of one area of forest can lead to increased destruction elsewhere, e.g. [38]) would mean that the extra layer of management specifically to protect the forest for orangutans would be less efficient. Both strategies would therefore be affected, although reintroduction would be affected less as it protects less forest area. There is not much quantitative work in Borneo on leakage, so it is hard to estimate the level of this effect at present. The current spatial variation of orangutan abundance depends on forest types and stages [20] and the degree of hunting. A future refinement of the model could take spatial variation in density into consideration, but we did not address this in the present study because there were insufficient data to analyze the extent to which density variation is caused by ecological conditions (e.g., food availability) or low threats (e.g., limited hunting pressure). Recent analyses [37] suggest that hunting pressure for food is generally high in areas with low orangutan densities, and low in areas with high densities. Other reasons for killing (e.g., crop conflict) are, however, often concentrated in areas of high threat and high densities. At the moment, we do not sufficiently understand the interplay between ecology, killing rates, and densities, making it difficult to incorporate this into our present model. However, the reintroduction strategy puts orangutans into unpopulated forest, so spatial variation in orangutan abundance is irrelevant for this strategy. A strategy that accounted for spatial variation in abundance would therefore only increase the benefits to the protection strategy.

Both Indonesia and Malaysia call for sustainable management of natural resources for the benefit of present and future generations of people. Conservation therefore needs to deal with time frames of 100 years or more. Our models suggest that protection of wild populations and their habitat is a better strategy over such time frames. Why then is reintroduction presently employed as a strategy for conserving orangutans?

Although conservation planning might be thought of as always long-term, the reality is that decisions are typically made to time frames that are often relatively short. Different groups value rewards over different time horizons and conservation funding often tends to be short-term in nature. For short time scales, our results showed a cost-efficient allocation of funds would be to fund rehabilitation and reintroduction of orangutans. There are also other benefits of orangutan rehabilitation centres: the survival and welfare of the reintroduced individuals; improved enforcement of wildlife protection laws and a solution for law enforcement when animals that are kept illegally are confiscated by relevant authorities; and increased public awareness about conservation [7]. Increased ability to raise conservation dollars could also be a significant benefit. Rehabilitation centres provide visible evidence of the impacts of poaching and habitat destruction, and a strongly emotional call for constructive conservation solutions. We recognise that funding available for orangutan rehabilitation and reintroduction can originate from different sources than the funding available for forest protection. Because some of the money available for orangutan conservation has a different origin and is made available for a different motivation, part of the funding allocated to rehabilitation will never be available for forest protection.

There are no reliable data on how much conservation funding is presently spent on orangutan reintroduction versus habitat protection, but we think overall funding (including government and non-government sources) is presently about equally divided between the two strategies. From an animal welfare perspective, rehabilitation centres are valuable. From a purely conservation point of view, however, funding should be allocated primarily towards habitat protection and management. If funding is fungible across the two strategies, the emotive aspects of rehabilitation programs can be counter-productive for the long-term conservation of wild orangutans.

The fact that the majority of wild orangutans currently live outside forests with conservation status [3] implies that we must develop innovative strategies to manage orangutan habitats in landscapes potentially threatened by silvi- and agricultural production. This requires long-term solutions for managing remaining habitats more sustainably [39] and addressing complex conservation issues at the landscape level. Well managed production forests using reduced impact logging techniques are one possible solution.

Insights from the present analysis provide conservation authorities and non-governmental organizations a more rational framework for prioritizing their investments to different strategies. Aside from providing important feedback to donors financing these activities, this information should help develop an improved integration between the organizations that implement these strategies and hopefully lead to optimal outcomes for orangutans and other users of forest services.

## Supporting Information

File S1 (DOC)
