# Supplementary material for: Conservation Strategies for Orangutans: Reintroduction versus Habitat Preservation and the Benefits of Sustainably Logged Forest
Source: PLoS One. 2014 Jul 15;9(7):e102174. doi: 10.1371/journal.pone.0102174 (PMC4099073; doi:10.1371/journal.pone.0102174)
Supplement: File S1 — (DOC) [file pone.0102174.s001.doc]

**SUPPORTING INFORMATION**

**Methods S1**

*1. Conservation forest only*: The simplest case assumes that, within the timescale studied, there is always enough conservation forest free of orangutans for reintroduction. Consequently, we don’t need to model the dynamics of orangutan-free forest. Additionally, only orangutan inhabited forest is protected (via P or R), so there is also no need to model orangutan-free protected forest. Hence we have the two state model in the main paper:

$\begin{matrix} \frac{dCF}{dt}=-d_{1}CF-\gamma\alpha_{1} \\ \frac{dCF_{P}}{dt}=-(1-e_{1})d_{1}CF_{P}+\gamma\alpha_{1}+(1-\gamma)\beta\end{matrix}$ (S1)

The total number of hectares of forest populated by orangutans at time *t, N(t),* is found by integrating model (S1) with respect to time and summing *CF* and *CF_p_*:

$N(t)=CF(0)e^{-d_{1}t}-\frac{\gamma\alpha_{1}}{d_{1}}(1-e^{-d_{1}t})+\frac{(\gamma\alpha_{1}+(1-\gamma)\beta)}{d_{1}(1-e_{1})}(1-e^{-d{}_{1}(1-e_{1})t})$ (S2)

The strategy that maximises the value of *N(t)* at time *t* can be determined by differentiating equation (S2) with respect to *γ*. As this is linear with respect to *γ*, then the optimal solutions will occur at the limiting values for *γ*. For small *t*, the optimum solution is *γ* *= 0* (R), and for large *t* the optimum is *γ = 1* (P) when calculated using the parameters in table 2 in the main text. The point in time, *t_c_*, when we switch from one strategy to the other can be found by solving either *dN(t) / dγ = 0* for *t* (or equivalently solving N(t, *γ=0) N*(t, γ=1) for *t*). This equation is transcendental, but an approximate solution can be found:

$t_{c}=\frac{2\beta}{d_{1}\alpha_{1}-d_{1}(1-e_{1})(\alpha_{1}-\beta)}+O(d_{1}t)^{3}$ (S3)

Substituting for α_1_ and β:

$t_{c}\approx\frac{2C_{P}}{d_{1}C_{R}-d_{1}(1-e_{1})(C_{R}-C_{P})}\approx\frac{2}{d_{1}e_{1}}\frac{C_{P}}{C_{R}}$, when *C_R_>> C_P_* (S4)

*C_P_* will always be smaller than *C_R_* as *C_P_* is the cost of protection, whereas the reintroduction budget includes the much larger cost of rehabilitation plus the cost of protection (see Table 2, main paper). For time horizons shorter than *t_c_*, the optimal strategy is to spend the entire budget on re-introducing orangutans (R). For time horizons larger than *t_c_*, the optimal strategy is to spend on protecting conservation areas (P).

*2. Conservation and timber production forest*: The simple model can be extended (as in the main paper):

$\begin{matrix} \frac{dCF}{dt}=-d_{1}CF-\gamma(1-\theta)\alpha_{1} \\ \frac{dTP}{dt}=-d_{2}TP-\gamma\theta\alpha_{2} \\ \frac{dCF_{P}}{dt}=-(1-e_{1})d_{1}CF_{P}+\gamma(1-\theta)\alpha_{1}+(1-\gamma)\beta\\ \frac{dTP_{P}}{dt}=-(1-e_{2})d_{2}TP_{P}+\gamma\theta\alpha_{2} \end{matrix}$ (S5)

The parameter *θ* changes the proportion of the P budget spent on conservation or timber production forest. We are interested in finding the optimal value of the parameter *θ* that maximises the total amount of orangutan habitat (for the case when γ > 0, as γ = 0 means the entire budget is spent on reintroduction, in which case the strategy *θ* is irrelevant). The total amount of orangutan habitat over these forest types, *N(t)*, is given by integrating equation (S5) (the initial amounts of protected forest are zero) and summing *CF*, *φTP*, *CF_p_* and *φTP_p_,* where *φ* is the density of orangutans in timber production forest relative to conservation areas (0 < *φ* < 1):

(S6)

The optimal protection strategy *θ* that maximises the value of *N(t)* at time *t* can be determined by differentiating *N(t)* with respect to *θ* :

, assuming O(d_i_ t^3^) ≈ 0 (S7)

So that if α_1_*d_1_e_1_>φ α_2_d_2_e_2_*, then *dN(t)/dθ < 0* and the optimum strategy is*θ* = 0, i.e. protecting conservation areas is a better strategy than managing and protecting timber production forest and all the resources are directed to protecting conservation areas (assuming *CF(t) > 0*). Dividing each side of the inequality by the total budget per year, then the strategy *i* that maximises *φ_i_ d_i_ e_i_* / *C_i_* is optimal (*φ_i_* = 1 for conservation forest).

**Assumptions**

*There will always be enough conservation forest free of orangutans for reintroduction*. Based on our estimates of the cost of rehabilitation, a $1M annual budget would reintroduce 17.4 orangutans per year and protect 736 ha of forest. Our current estimate is that there is over 6 million ha of orangutan-free conservation area forest on Borneo. Even over a 100 year timescale, incorporating current rates of forest loss, and assuming much higher budgets, there will always be enough of this forest for reintroduction. We therefore think this is a valid assumption.

*There are always enough orangutans in rehabilitation centres for reintroduction.* Based on current numbers in centres of over 1,000 orangutans, and assuming only a small percentage of orangutans potentially lost due to habitat destruction come into rehabilitation centres, then at 17.4 orangutans reintroduced per year ($1M annual budget) it is a reasonable assumption that there will always be enough orangutans in centres to be reintroduced.
